# From Trial to Chronic Epidural Electrical Stimulation: A Single-Patient Feasibility Study in Motor-Complete Spinal Cord Injury

**DOI:** 10.3390/jcm15176663

**Published:** 2026-08-28

**Authors:** Alena Militskova, Elvira Mukhametova, Artur Biktimirov, Vyacheslav Andrianov, Elena Yakovleva, Dinara Silantyeva, Igor Lavrov

**Affiliations:** 1 Federal Center of Brain Research and Neurotechnologies, Federal Medical Biological Agency of Russia, Moscow 117513, Russia; milickova@fccps.ru (A.M.);; 2 Life Improvement by Future Technologies (LIFT) Center, Moscow 121205, Russia; 3 Institute of Fundamental Medicine and Biology, Kazan Federal University, Kazan 420008, Russia; 4 Department of Biomedical Engineering, Mayo Clinic, Rochester, MN 55905, USA; 5 Department of Neurology, Mayo Clinic, Rochester, MN 55905, USA; 6 Department of Radiology, Mayo Clinic, Rochester, MN 55905, USA

**Keywords:** spinal cord injury, motor-complete SCI, epidural spinal cord stimulation, voluntary control, spinally evoked motor potentials, diagnostics, neuromodulation, neuroplasticity

## Abstract

**Background/Objectives:** Epidural electrical stimulation (EES) is a valuable intervention for motor recovery after spinal cord injury (SCI). However, evaluating expected outcomes can be challenging due to the difficulty of assessing residual functional connectivity and other key factors underlying the effects of EES. This case study investigates EES with two 8-contact percutaneous electrodes (Perc-EESs) as a prognostic tool and therapeutic bridge for chronic neuromodulation with EES in an individual with clinically motor-complete SCI. **Methods:** A 40-year-old male (AIS-B, Th5–Th6, >20 years since initial presentation followed by a complex history of progressive deterioration and two subsequent neurosurgical interventions) underwent a 7-day trial using Perc-EES, followed by chronic 5-6-5 Medtronic lead (Paddle-EES) implantation at the L2–S1 segments. Volitional motor control and EMG activity was assessed across three stages: Perc-EES at 5, 7, and 10 days post-enrollment; Paddle-EES at 13, 15, and 18 days post-enrollment; and at a 1-year follow-up (Paddle-EES (1y)). Evaluations were performed in side-lying and upright positions. **Results:** Without EES, no visible movements were observed. Perc-EES facilitated rhythmic EMG activity and initial voluntary movements, providing an early proof-of-concept that Perc-EES could serve as a preliminary assessment of the patient’s responsiveness to neuromodulation. Paddle-EES (1y) demonstrated a further improvement in motor coordination. Analysis using the Rudolph coefficient showed a distinct shift toward zero. This shift reflects stimulation-associated changes in EMG coordination, moving away from co-activation toward a more alternating activation pattern across side-lying and upright positions. **Conclusions:** This single-patient feasibility case study suggests that trial Perc-EES may have potential in determining the long-term benefits of chronic Paddle-EES in motor activation after SCI. While these conclusions remain preliminary, these findings can further serve as a potential diagnostic tool to optimize electrode placement and enhance overall recovery in motor-complete SCI patients.

## 1. Introduction

Spinal cord stimulation has been successfully implemented to control chronic pain and improve neurological disorders, including paralysis and non-motor symptoms following SCI [1,2,3,4,5,6,7,8]. Recent advances in neuromodulation have raised hopes for new therapeutic approaches to treat SCI. Electrical spinal cord stimulation has shown significant improvements in patients with both complete and incomplete SCI, reducing spasticity and facilitating voluntary movements, independent standing, balance, and improved bowel and bladder control [9,10,11,12,13,14,15,16,17,18,19]. However, several concerns limit the clinical implementation of epidural electrical stimulation (EES) for SCI therapy, including efficacy (92.9%), lack of clear guidelines on stimulation parameters (83.3%), and difficulty in identifying which patients will benefit from EES (76.2%) [20].

EES is hypothesized to enable supraspinal signaling via residual neural pathways crossing the lesion, facilitating sublesional spinal circuitry [1,21]. The ability to regain neural control below the injury level in patients with motor-complete SCI is often referred to as a discomplete type of SCI [10,22]. Identifying residual fibers is a complex task and requires instrumental assessment applied alone or combined with functional tests and therapeutic strategies, such as motor training and neuromodulation [23]. EES paired with motor rehabilitation significantly improves voluntary movements [22,24], which may persist even without ongoing stimulation [25]. This suggests that motor training is critical in preparing spinal circuitry to respond to EES. However, recent reports also indicate improvements in voluntary muscle activity with EES alone, without rehabilitation [26].

Early anatomical studies showed that many subjects with clinically complete SCI have anatomical connectivity across the injury [27]. Importantly, current clinical and instrumental assessments for SCI are not sensitive enough to identify the functional role of these fibers [28]. Consequently, patients with clinically complete SCI can be diagnosed as ‘discomplete’ only by incidental evidence of translesional connectivity, often demonstrated outside routine clinical protocols [23,29]. In this context, percutaneous-EES (Perc-EES) demonstrated feasibility and acute responsiveness to trial stimulation in an SCI patient prior to chronic Paddle-EES implantation. Most studies on volitional control with EES after SCI have used chronically implanted paddle leads [12,26,30,31], while Perc-EES typically employs percutaneous rod electrodes. A direct comparison of the effects of both electrode types is warranted, given the higher migration rate of rod electrodes [32] and the different contact surfaces with the dura mater [33,34,35,36,37].

Here, we hypothesized that trial epidural spinal cord stimulation with Perc-EES may help assess acute responsiveness to epidural spinal cord neuromodulation prior to chronic Paddle-EES implantation aimed at modulating spinal circuitry and restoring neurological function. This report describes a participant diagnosed with motor-complete SCI (AIS B) due to multiple intramedullary tumor resections. Paralyzed for over ten years and without receiving regular rehabilitation, the participant was enrolled in this study and tested without motor rehabilitation to assess the effect of EES. Volitionally initiated and controlled stepping-like patterns were generated first with a trial percutaneous electrode and later with a chronic paddle electrode. The effect of EES was assessed in both side-lying and upright positions on a treadmill with body weight support (BWS). A notable outcome of this study is the immediate improvement in motor and non-motor functions during Perc-EES, which was maintained and further improved during Paddle-EES (1y).

## 2. Case Presentation

**Figure 1:**
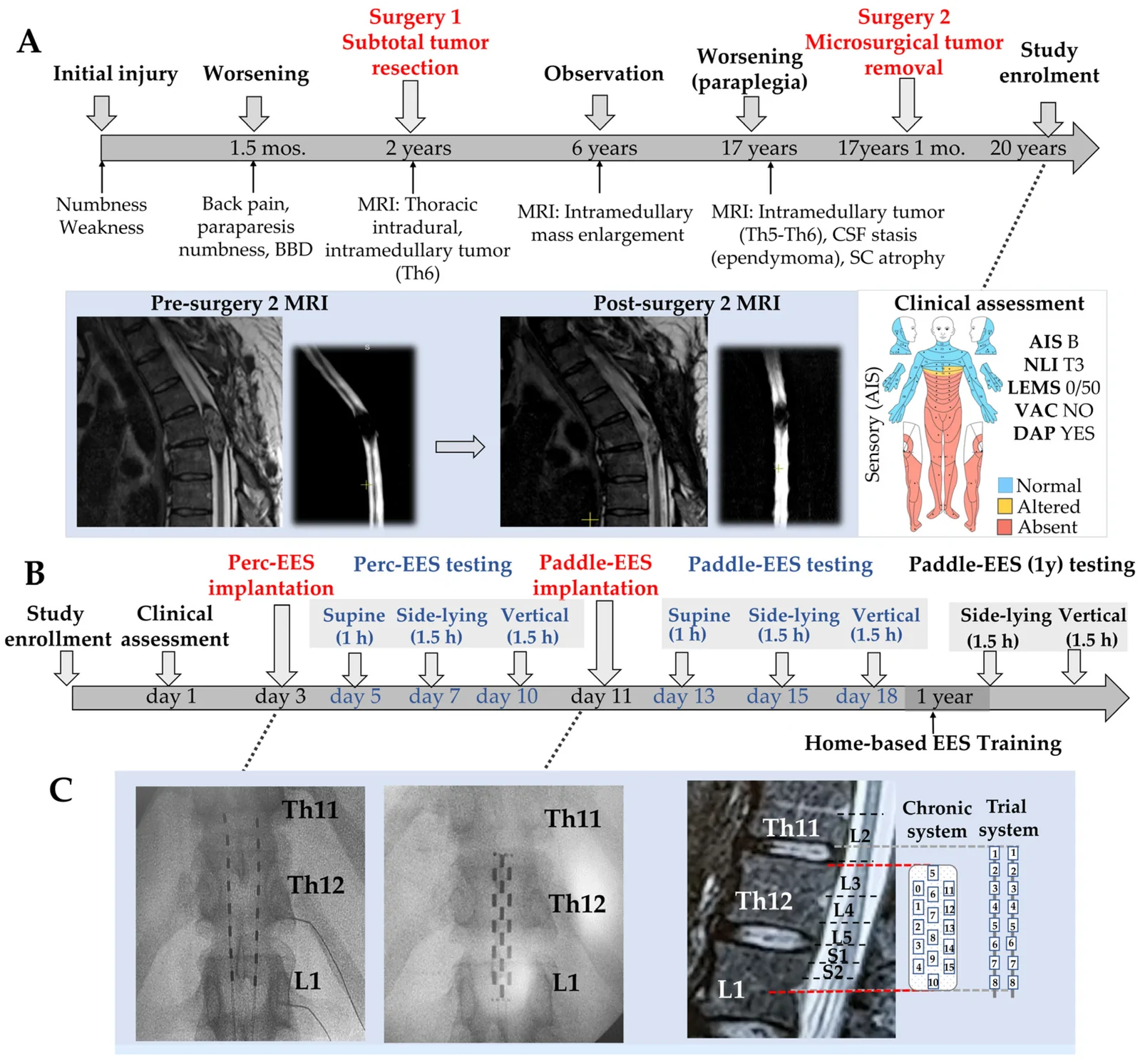
Clinical history and study timeline. (**A**) Overview of the patient’s medical history. Key interventions include: prednisolone pulse therapy and two surgical procedures: surgery 1 (subtotal tumor resection, two years after symptoms onset) and surgery 2 (microsurgical tumor removal, 2016). MRI findings (right panels) show the spinal cord at the Th5–Th6 segment. The pre-surgery MRI (17 years after symptoms onset and symptoms worsening: paraplegia) and post-surgery MRI (18 years after symptoms onset) demonstrate stable atrophic changes in the spinal cord without signs of progressive growth. (**B**) Study timeline and experimental design. Clinical assessment included the ISNCSCI protocol and questionnaires (quality of life, mental health, and bowel, bladder, and sexual functions). Electrophysiological testing with EMG registration during voluntary movement attempts and epidural electrical stimulation (EES), including Pecr-EES, Paddle-EES testing and Paddle-EES (1y) testing phases, enabled training and assessment in three positions: supine, side-lying (with the top leg suspended), and upright (in a body weight support system). (**C**) Electrode localization. X-ray images showing the percutaneous lead position during trial (Perc-EES) (implanted at the Th11–L1 level) and the permanent paddle electrode array (Paddle-EES) (Medtronic 5-6-5) lead position. The right panel displays a zoomed CT scan of the lumbar spinal cord with corresponding spinal segments (L2–S1) and the correlated electrode position relative to the lumbosacral locomotor circuitry. The red color on the timeline indicates electrode implantation and surgical procedures. BBD—bladder and bowel disorder; MRI—magnetic resonance imaging; CSF—cerebrospinal fluid; ISNCSCI—International Standards for Neurological Classification of Spinal Cord Injury; EMG—electromyography; EES—epidural electrical stimulation; CT—computed tomography: AIS—ASIA Impairment Scale; NLI—neurological level of injury; LEMS—lower extremity motor score; VAC—voluntary anal contraction; DAP—deep anal pressure.

This case report describes a 40-year-old man (height of 167 cm/65.75 inches, body weight of 80 kg/176 lb) with a spinal cord injury of mixed ethiology (AIS-B: sensory-incomplete, but motor-complete). Twenty years before enrollment, following a minor injury to his thoracic spine, he noticed weakness in both legs (left > right) and numbness. About 1.5 months later, he also developed mid-thoracic back pain, further leg weakness, and bladder and bowel dysfunction (voiding hesitancy and straining). Neurological examination at that time showed lower-limb paraparesis 3–4/5, hypoesthesia below the Th6–Th7 level bilaterally, and voiding difficulty. Over the following months the symptoms progressed with increasing weakness and worsening gait. Two years after symptom onset, a spinal cord MRI revealed an intradural, intramedullary tumor at Th6 (26 × 7.5 mm), and a subtotal resection was performed (Surgery 1; Figure 1A), with histology confirming ependymoma. No further changes were seen on follow-up MRIs until 4 years later (6 years after symptom onset), when imaging showed tumor enlargement (35 × 9 mm) compared with the previous study. The patient declined the recommended second operation. Over the following years he developed progressive ambulatory impairment and gradually became non-ambulatory. He presented again 11 years later (17 years after symptom onset) with progressive worsening of symptoms, increased spasticity, and severe back pain. Neurological examination at that time showed lower-limb paraplegia with constipation and urinary retention. Spinal cord MRI showed an intramedullary tumor (32 × 14 mm) at Th5–Th6 with signs of CSF stasis. He underwent a second resection of the intradural, intramedullary ependymoma (Surgery 2; Figure 1A), with subsequent pain relief.

At enrollment (>20 years after initial symptom onset), the participant had been paraplegic (AIS B) for approximately 4 years. During the 3 years following the second surgery he received 3 courses of inpatient rehabilitation (3 weeks each), with initial gains that enabled him to stand with support and increased muscle strength in the back and upper extremities; the last course was completed 10 months before enrollment. His neurological status had been clinically stable throughout this period.

The participant met the inclusion criteria: complete paraplegia (AIS B) at Th1–Th12 levels of non-traumatic etiology, presence of spinal reflexes, and absence of other acute or chronic comorbidities. He also met the exclusion criteria: no lower motor neuron injury, no severe spasticity (>2 on the Modified Ashworth Scale), no active urinary tract infection, time since injury >12 months, no skin problems, and no history of other neurological, psychiatric, cognitive, or orthopedic conditions. Participant was selected as a clinically informative case combining several features: non-traumatic mixed etiology (chronic myelopathy secondary to intramedullary ependymoma with a post-surgical component from two resections), chronicity of more than 20 years from symptom onset with approximately 4 years of stable complete paraplegia, and the absence of ongoing supervised rehabilitation at enrollment. This profile placed participant at the more complex and less studied end of the SCI spectrum, and made participant a suitable candidate for evaluating the effect of trial EES prior to the decision on Paddle-EES implantation, and subsequently the effect of chronic EES combined with a structured home-based, stimulation-assisted rehabilitation program, against a long, stable neurological baseline.

After providing informed consent, the participant entered the study protocol, which consisted of trial percutaneous-EES implantation with two 8-contact leads (Octrode model 3086; Abbott, Plano, TX, USA), followed by chronic paddle-electrode implantation with a 16-contact epidural array (Specify 5-6-5; Medtronic, Fridley, MN, USA), and a 1-year home-based rehabilitation program enhanced by stimulation (Figure 1C). Both EES systems were positioned under fluoroscopic and electrophysiological guidance to ensure precise placement (Sf). Assessments were collected at baseline: on days 1, 5, 7, and 10 (Perc-EES testing); on days 13, 15, and 18 (paddle-electrode testing); and 1 year after paddle-electrode implantation (Figure 1B). Assessments comprised clinical and instrumental methods, including neurological examination and the following scales and tests: the International Standards for Neurological Classification of Spinal Cord Injury (ISNCSCI), the Spinal Cord Independence Measure (SCIM III), the Neurogenic Bowel Dysfunction Score (NBDS), the Neurogenic Bladder Symptom Score (NBSS), the Patient Health Questionnaire-9 (PHQ-9), the Short Form-36 (SF-36), and the Modified Ashworth Scale (MAS) (Supplementary Tables S1 and S2).

Electromyography (EMG) signals were obtained bilaterally from the rectus femoris (RF), vastus lateralis (VL), biceps femoris (BF), tibialis anterior (TA), medial gastrocnemius (GM), and soleus (SOL) muscles, using LabChart 8 software and a Bioamp/PowerLab system (ADInstruments, Inc., Dunedin, New Zealand). Signals were filtered with a 50 Hz notch filter and a 20–1000 Hz bandpass filter, and sampled at 4 kHz. EMG analysis included the latency and peak-to-peak amplitude of spinally evoked motor potentials (SEMPs) for intraoperative monitoring (Supplementary Figure S1), peak-to-peak amplitude to evaluate voluntary movement activity, and, for rhythmic activity (10–20 patterns), the area under the curve (AUC) calculated in the EMG signal of each muscle, together with the reciprocal activity of antagonist muscles (Rudolph coefficient).

Analyses were performed using OriginPro 2021 (OriginLab Corporation, Northampton, MA, USA) (more informative descriptions of procedures available in Supplementary Materials File S1).

## 3. Results

In this study, we performed a comprehensive evaluation of functional and neurophysiological outcomes across three distinct stages: Perc-EES using an Abbott percutaneous system (5, 7, and 10 days post-enrollment); Paddle-EES using a Medtronic 5-6-5 surgical lead (13, 15, and 18 days post-enrollment); and Paddle-EES (1y) using the Medtronic 5-6-5 system at 1-year follow-up. The assessment was focused on monitoring voluntary motor control of the lower limbs and characterizing EES-facilitated locomotor activity. To quantify quality of movements and motor coordination, we used electromyography (EMG) assessment and calculated the Rudolph coefficient and the area under the curve (AUC) for key metrics. A critical component of the study was the investigation of the impact of body position during training sessions on stimulation efficacy, comparing stimulation parameters and outcomes in the side-lying position (EES-facilitated step-like activity) versus upright stepping in BWS. In addition to motor performance, dynamic neurological assessments were conducted using the ISNCSCI scale and monitored pelvic organ functions via the Neurogenic Bladder Symptom Score (NBSS). Our findings indicate that trial stimulation with Perc-EES is feasible and elicits acute neuromodulatory responses, supporting its potential role as a preliminary assessment prior to chronic Paddle-EES implantation. Further studies on larger cohorts are needed to confirm whether acute responsiveness to Perc-EES may predict long-term outcomes with Paddle-EES.

### 3.1. Motor Performance with Trial Perc-EES and Paddle-EES During “Flexion–Extension” Test

**Figure 2:**
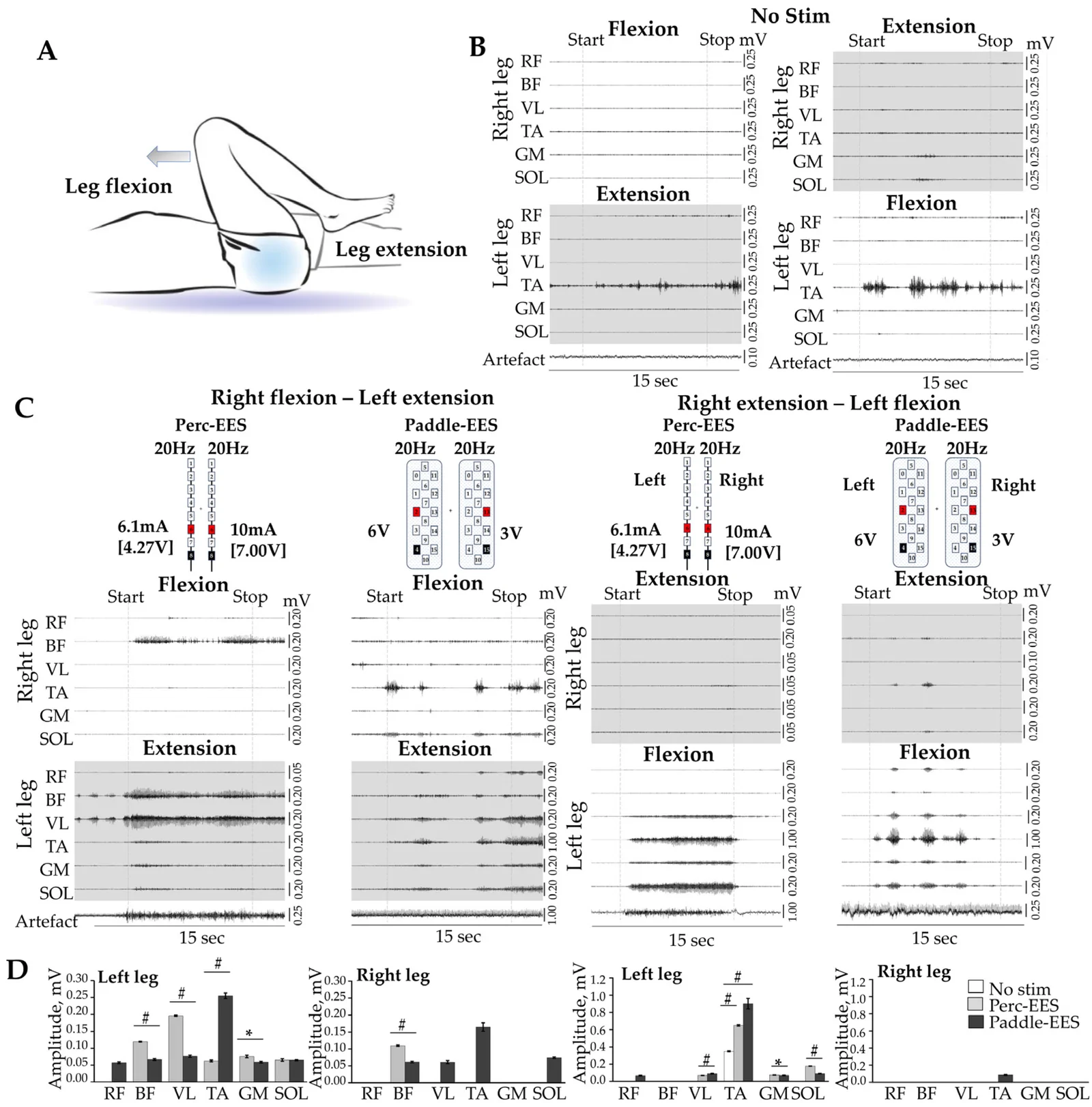
EES-enabled voluntary motor activity during leg flexion–extension test. (**A**) Body positioning during the flexion–extension test: the subject is in a supine position with the limb supported on the table surface. The arrow indicates the direction of leg movement. (**B**) Representative EMG recordings of lower extremity muscles during volitional attempts to perform the flexion–extension test without stimulation (no stim). Vertical dashed lines indicate the onset and termination of verbal commands. White background records indicate EMG recorded during leg flexion; gray background records indicate EMG recorded during leg extension. (**C**) Schematic visualization of stimulation protocols and electrode configurations: Perc-EES (contacts 6+8−, 20 Hz + 20 Hz) and Paddle-EES (contacts 3+4− and 14+15−, 20 Hz + 20 Hz) applied bilaterally. Representative EMG recordings of lower extremity muscles during volitional attempts to perform the flexion–extension test during Perc-EES and Paddle-EES. Red and black colors on the electrode image designate the anode and cathode, respectively. (**D**) Quantitative comparison of EMG peak-to-peak amplitudes during voluntary movements. Data represents Perc-EES (light gray) and Paddle-EES (black) stages, and no stim (white). RF—m. rectus femoris; VL—m. vastus lateralis; BF—m. biceps femoris; TA—m. tibialis anterior; GM—m. gastrocnemius medialis; SOL—m. soleus. * *p* < 0.05; # *p* ≤ 0.001.

Volitional movements in both legs were initially assessed along with EMG activity in a supine position and then in a side-lying position with the upper leg suspended and freely moving. Command-associated activity was evaluated by its temporal alignment with verbal instructions, trial-to-trial reproducibility, verified onset/offset, and directional changes relative to non-stimulation periods. A baseline three-minute relaxation period preceded testing to screen for spontaneous or spastic activity. Figure 2A illustrates the body positioning during the flexion–extension test, where movements were performed with the limb supported on the table surface. Representative EMG traces of the lower extremity muscles during the flexion–extension test performed without EES (no stim) are presented in Figure 2B, and with EES in Figure 2C, where dashed lines indicate the onset and termination of verbal commands. Figure 2C shows the stimulation protocols used during voluntary control attempts during trial electrical stimulation (Perc-EES), and electrical stimulation with paddle electrodes (Paddle-EES), with specific electrode configurations. Attempts to perform the “flexion–extension” test without EES did not produce visible movement (Supplementary Video S1). EMG analysis revealed only spontaneous muscle activity in the left tibialis anterior (TA), unrelated to the verbal commands (Figure 2B). In contrast, voluntary efforts during Perc-EES elicited tonic muscle activity, primarily in the left leg, in response to the commands. During Paddle-EES, the EMG activity was observed in most muscle groups across both legs, occasionally manifesting as rhythmic activity (Figure 2C). Notably, during the initial Perc-EES session, while the participant could move the left foot in the correct direction, his right foot moved in the opposite direction of the command (Supplementary Video S1A). However, after 2–2.5h of EES-facilitated training and visual feedback, the participant successfully generated voluntary right-foot movements in accordance with instructions (Supplementary Video S1B). These findings might suggest the rapid plasticity of the spinal circuitry in mediating supraspinal volitional commands facilitated by EES.

Quantitative analysis of EMG amplitudes during the flexion–extension test is summarized in Figure 2D. Without EES, no visible movements were observed, and no distinct EMG activity was detected across the recorded muscles, except for residual baseline activity captured in the left TA during the attempted movement of ‘right flexion–left extension’ (white bars). In the case of the EES-facilitated right leg flexion, m. biceps femoris (BF) activity was present in both trial Perc-EES (light gray bars) and Paddle-EES (black bars) stages. However, recruitment of the VL, TA, and SOL emerged exclusively during the Paddle-EES. In the case of the right leg extension, activation of the right TA was recorded above the threshold of 0.05mV only during Paddle-EES (black bar). In the case of the left leg extension, the m. rectus femoris (RF) was recruited exclusively with Paddle-EES, whereas the BF, VL, TA, GM, and SOL were active during both trial Perc-EES and Paddle-EES. Distinct intra-individual variations in EMG amplitudes between trial and Paddle-EES were identified in the BF, VL, TA, and GM (all *p* < 0.01). In the case of the left leg flexion, the RF was also recruited only with Paddle-EES. The VL, TA, GM, and SOL exhibited activity during both trial Perc-EES and Paddle-EES, with substantial changes in EMG amplitudes observed in the VL, TA, GM and SOL (all *p* < 0.01) (Figure 2D). Overall, while the participant remained unable to generate visible movement without EES, both trial Perc-EES and Paddle-EES facilitated volitional control over lower-limb EMG activity. Detailed EMG amplitude values are provided in Supplementary Materials, Table S3.

### 3.2. Trial Perc-EES and Paddle-EES Facilitated Rhythmic Activity in Side-Lying Position

**Figure 3:**
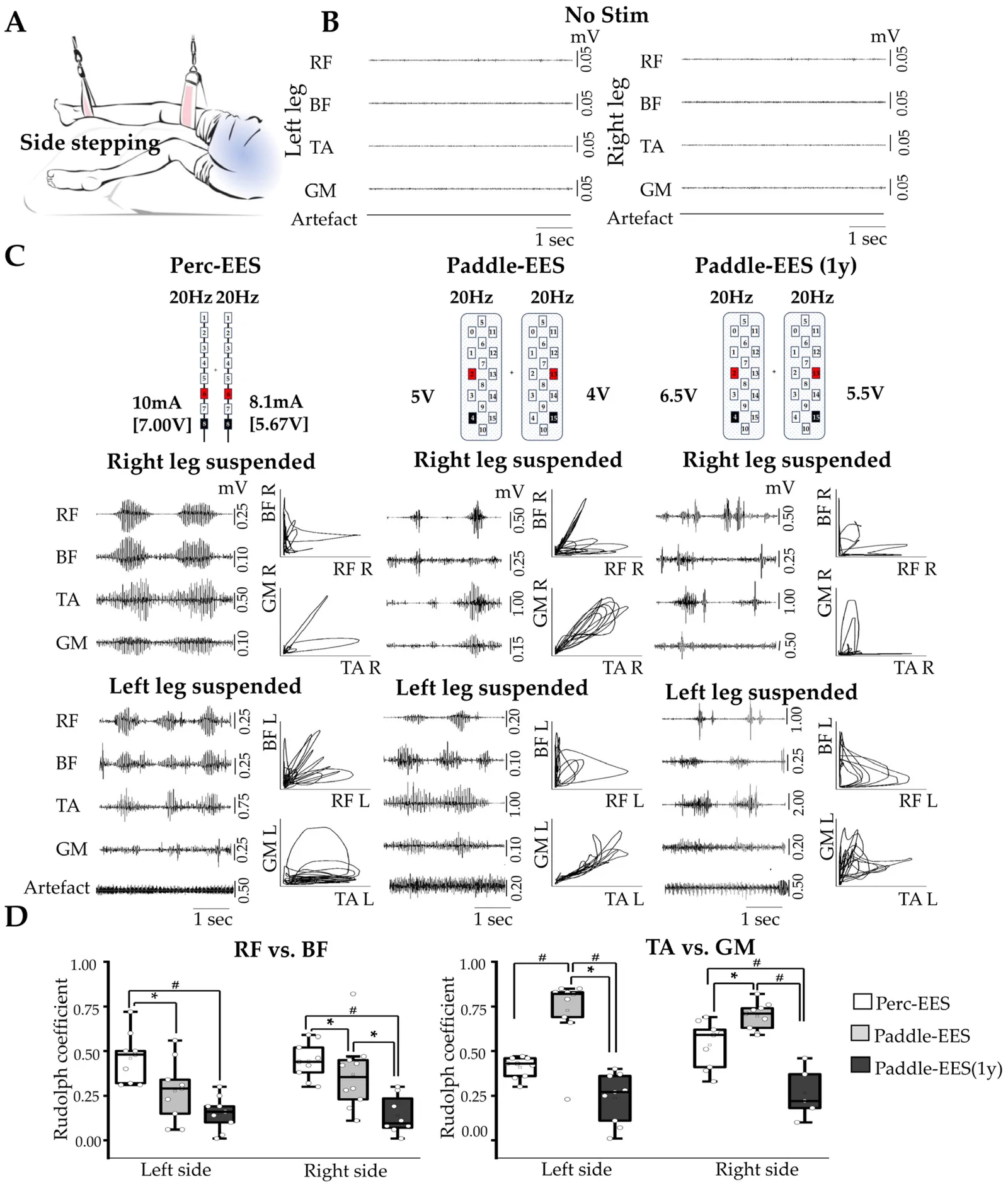
EES-facilitated rhythmic activity in: (**A**) subject positioning in a suspension system designed to unload the limb for side-lying assessment. (**B**) Representative EMG recordings during voluntary attempts to step in the absence of stimulation (no stim). (**C**) EES-enabled rhythmic patterns across Perc-EES, Paddle-EES, and Paddle-EES (1y) in side-lying position. Specific electrode configurations are indicated: 6+8− (Perc-EES); 3+4−/14+15− and 2+4−/13+15− (Paddle-EES, Paddle-EES (1y)). Phase diagram inserts (RF vs. BF; TA vs. GM) illustrate the evolution toward an L-shaped trajectory. Red and black colors on the electrode image designate the anode and cathode, respectively. (**D**) Statistical analysis of coordination in side-lying position: Rudolph coefficient values showed a distinct shift toward zero during Paddle-EES (1y). * *p* < 0.05; # *p* ≤ 0.001; ° indicates individual data points.

Figure 3A shows the subject’s positioning in a suspension system designed to unload the limb. In the absence of electrical stimulation (no stim), no EMG activity was recorded during voluntary attempts to step (Figure 3B). In contrast, the application of EES enabled the generation of the volitional rhythmic motor patterns. The specific electrode configurations and corresponding EMG recordings during different phases of EES are shown in Figure 3C. The modulation of EMG activity was clearly dependent on the stimulation site and intensity. Thus, caudal stimulation (around L5-S1) using contacts 6+8− (Perc-EES) and 3+4−/14+15− (Paddle-EES) yielded the optimal motor outcomes for side-lying stepping, resulting in robust rhythmic EMG signals and visible limb movements (Supplementary Video S2). The motor threshold for volitionally induced EMG activity in a side-lying position was 5.8 ± 1.22 V (8.25 ± 1.75 mA; 700 Ω) during Perc-EES and 5.0 ± 0.9 V during Paddle-EES. The primary immediate effect of both EES modalities (Perc-EES and acute Paddle-EES) was the facilitation of volitional control and the initiation of lower-limb rhythmic activity. In contrast, long-term assessments at one year revealed a distinct transition toward alternating muscle coordination patterns, demonstrated by the Rudolph coefficient shifting toward zero and phase diagrams evolving into L-shaped trajectories (Figure 3D). Rudolph coefficient values near zero indicated asynchronous muscle activity, whereas values near one reflected pronounced co-contraction. The coordination of muscle pairs (RF vs. BF and TA vs. GM) was evaluated using phase diagrams (Figure 3C, EMG inserts). Comparative analysis, summarized in Figure 3D, revealed that while distinct shifts were evident during the transition from trial Perc-EES to Paddle-EES (1y) (e.g., left TA vs. GM, *p* ≤ 0.001), the most visible recovery occurred during the Paddle-EES (1y) compared to Perc-EES (*p* < 0.001). Detailed EMG coordination values are provided in Supplementary Materials, Table S4.

### 3.3. Trial Perc-EES and Paddle-EES Facilitated Rhythmic Activity in Upright Position

**Figure 4:**
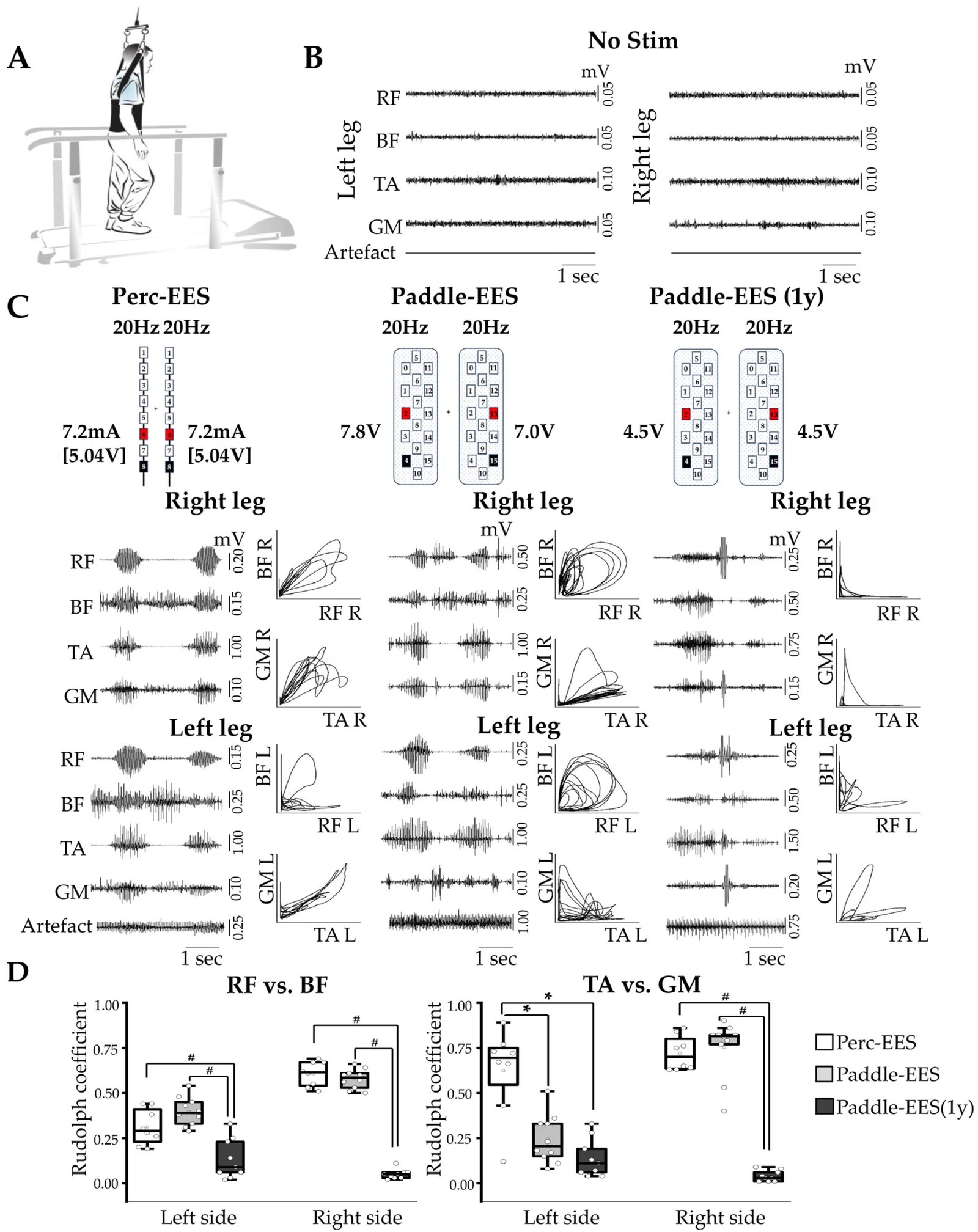
EES-facilitated rhythmic activity in upright position. (**A**) Subject positioning in the upright position using a body weight support (BWS) system. (**B**) EMG recordings in the upright position without stimulation (no stim). (**C**) EES-facilitated rhythmic activity during treadmill locomotion (~60% unloading). Phase diagram inserts demonstrate the transition toward characteristic L-shaped trajectories for antagonistic proximal and distal muscles. Red and black colors on the electrode image designate the anode and cathode, respectively. (**D**) Statistical analysis of coordination in the upright position: averaged Rudolph coefficients for antagonistic muscle pairs show significant reductions in co-activation: * *p* < 0.05; # *p* ≤ 0.001; ° indicates individual data points. EMG collected bilaterally from: rectus femoris (RF), biceps femoris (BF), tibialis anterior (TA), and gastrocnemius medialis (GM).

Further evaluation was conducted in an upright position using a body weight support (BWS) system (Figure 4A). The motor threshold for EES-induced rhythmic activity in upright position was 6.4 ± 1.1 V during Perc-EES, 6.5 ± 0.8 V during Paddle-EES, and 4.8 ± 0.6 V during Paddle-EES (1y) (impedance: 850 Ω). Similar to the side-lying condition, no EMG activity was observed without EES (Figure 4B). Under ~60% body weight unloading on a treadmill (0.2 m/s), EES facilitated consistent rhythmic activity across all tested stages (Figure 4C; Supplementary Video S3).

While the overall rhythmic activity remained stable, the EMG patterns of antagonistic muscle pairs underwent distinct changes after one year of EES. Phase diagram analysis showed a transition toward distinctive L-shaped trajectories for proximal and distal antagonist pairs (Figure 4C, L-shape inserts), indicating enhanced intra-limb coordination. The Rudolph coefficients for the upright position are presented in Figure 4D, with detailed statistics in Supplementary Table S4.

In contrast to the side-lying position, where the transition from Perc-EES to Paddle-EES improved muscle coordination in both the proximal (RF vs. BF: left *p* = 0.017, right *p* = 0.027) and distal leg muscles (TA vs. GM: left *p* < 0.001, right *p* = 0.018) (Figure 4C,D), the same transition in the upright position did not show any improvements (all *p* > 0.05) (Figure 4C,D). The effect of transitioning from Perc-EES to Paddle-EES yielded no distinct changes in upright muscle coordination patterns. However, long-term assessment at one year revealed a different outcome, marked by an essential improvement in locomotor patterns and a reduction in muscle co-activation. However, the transition to Paddle-EES (1y) led to a distinct shift in locomotor profiles, characterized by a lower level of co-activation in the left RF vs. BF, right RF vs. BF, and left TA vs. GM muscle pairs. Long-term assessment showed a more alternating, phase-dependent activation of leg muscles during these sessions, pointing to the cumulative effects of neuromodulation and intensive weight-bearing training. This indicates that while the trial phase demonstrates the immediate acute effects of EES, long-term stimulation-associated changes in EMG coordination are progressively established through sustained stimulation combined with rehabilitation.

Along with the coordination analysis, we quantified the magnitude of muscle recruitment by calculating the area under the curve (AUC) of the rectified EMG in both side-lying and upright positions (Supplementary Figure S2). Unlike the coordination metrics, the AUC results were characterized by high inter-muscle variability and heterogeneity across different stages of the study. The lack of systemic changes in EMG magnitude suggests that the long-term modifications were primarily driven by shifts in muscle coordination and alternating activation patterns, as reflected by the Rudolph coefficients, rather than a uniform increase in overall muscle activation.

### 3.4. Clinical Assessment

Autonomic bladder initiation occurred as an immediate effect during the initial EES trial and remained stable throughout the study. In contrast, sensory improvements and a shift in the neurological level of injury from T3 to T4 were detected only at the one-year evaluation. ASIA examination did not show changes in motor and sensory sub-scores a month after implantation. However, a year after implantation, clinical examination demonstrated both sensory and motor improvements: ISNSCI light touch sensory sub-scores increased from 45 to 47 and pin prick sensory sub-score from 45 to 48, and neurological level of injury changed from T3 to T4. AIS grade did not change throughout the study. At the same time, the participant reported a subjective facilitation of voiding initiation during the initial EES trial phase. This initial improvement was consistent one year after implantation. The Neurogenic Bladder Symptom incontinence sub-score changed from eight to seven to seven, storage-voiding sub-score from 12 to nine to eight, consequence sub-score from five to five to two and quality of life from two to two to one. For all domains, a higher score represents a worse symptom burden. The Neurogenic Bowel Dysfunction Score demonstrated a decrease by three points (from six to three to three), change in general satisfaction from eight (prior) to 10 (1m) to nine (1y) across the study (Supplementary Table S1).

To facilitate analysis, a comparative summary of clinical, motor, and electrophysiological outcomes between the two types of EESs (Perc-EES and Paddle-EES), alongside the long-term results at one year, is presented in Supplementary Table S5.

## 4. Discussion

Spinal cord injury presents significant clinical challenges due to the profound and often permanent loss of motor and sensory functions. One promising avenue for improving outcomes in SCI patients is the spinal cord neuromodulation with EES or transcutaneous stimulation. EES has been successfully used for decades to manage chronic pain, and Perc-EES is used for assessing the effect of spinal cord stimulation to control pain before implanting permanent leads [38]. EES application for neurological recovery in SCI demonstrated a great promise. This study aimed to compare the effects of Perc-EES and Paddle-EES (1y) on regaining volitional motor control in a participant with motor-complete SCI (AIS B). Volitional motor control and EMG activity were assessed across three distinct stages: Perc-EES using an Abbott percutaneous system (5, 7, and 10 days post-enrollment); Paddle-EES using a Medtronic 5-6-5 surgical lead (13, 15, and 18 days post-enrollment); and Paddle-EES (1y) using the Medtronic 5-6-5 system at 1-year follow-up. We hypothesized that Perc-EES could provide quick insights into acute motor responsiveness to EES in SCI patients, supporting its use as a feasibility and screening tool prior to chronic Paddle-EES implantation.

The participant, who had a long history of motor-complete SCI (AIS B), underwent consecutive implantation of trial and chronic electrodes, with intraoperative monitoring guiding electrode positioning. Two days post-implantation, upon the first activation of the Perc-EES system, the stimulation facilitated the immediate volitional movements below the injury level and also improved voiding initiation and emptying time. Paddle-EES demonstrated similar sustained effects on motor functions, maintained the bladder improvements, and additionally resulted in improved bowel function. Paddle-EES (1y) demonstrated similar effect on motor functions and maintained voiding initiation and emptying time. These findings are consistent with earlier reports suggesting that individuals with clinically motor-complete SCI (AIS B) may retain some functionality through residual neural pathways activated by EES [1,12,31,39]. Both trial Perc-EES and Paddle-EES (1y) elicited noticeable limb movements in the supine position following verbal commands, with corresponding coordinated EMG responses. Also, during the initial trial stimulation, the participant could follow commands with his left leg, but struggled with the right leg, which moved opposite to the intended direction. However, after 2–2.5h of EES-facilitated training with EES and visual feedback, the participant could correctly move both feet according to commands. The rapid emergence of volitional movement, rhythmic muscle activation, and improved voiding was observed in response to stimulation during both Perc-EES and Paddle-EES trials, without concurrent physical rehabilitation, and was more consistent with an immediate functional reconfiguration of spinal motor control mediated by sensorimotor feedback than with long-term structural plasticity. A notable finding in the flexion–extension test was the absence of rectus femoris (RF) EMG activity during Perc-EES that is contrasted with its activation during Paddle-EES. This discrepancy may reflect the biomechanical and structural differences in using dual percutaneous trial leads during dynamic movement. In the case of active flexion and extension, temporary percutaneous cylindrical leads can be susceptible to potential axial migration or micro-displacements within the epidural space as the patient changes positions [40]. Even when a dual percutaneous lead configuration was deployed, such minor instabilities might temporarily shift the active contacts away from the target upper lumbar segments (L2–L4) that innervate proximal motor pools like the RF [21]. Furthermore, unlike flat paddle leads designed to focus the electric current toward the spinal cord, cylindrical leads apparently tend to distribute energy more widely in all directions, which might partially reduce their target efficiency. Consequently, during task-specific testing, Perc-EES may not always maintain the same stable, targeted lateral field as the paddle lead [6]. Therefore, muscle activation profiles obtained during percutaneous trial stimulation should be interpreted with some caution, as these structural and stability differences can potentially limit the ability of Perc-EES to fully predict muscle responses.

In the side-lying position, both trial Perc-EES and Paddle-EES (1y) facilitated robust rhythmic movements, with co-activation of antagonist muscles in proximal and distal regions. This co-activation aligns with early physiological observations where tonic EES simultaneously recruits flexor and extensor networks prior to step-specific tuning [41]. When assessed in the upright position using the body weight support system, EES enabled stepping-like movements with reciprocal activity, confirmed that sensory proprioceptive feedback from limb loading and is critical to reorganize these coactive spinal circuits into functional, alternating motor outputs [2,12]. Although EES-enabled motor training led to more consistent alternating activation patterns over a one-year period, the AUC data showed high amplitude variability. This difference likely occurs because raw EMG amplitudes in patients with SCI are inherently highly variable. For instance, surface EMG signals can vary because of electrode placement, changing muscle fatigue, motor neuron excitability, fat thickness, skin temperature changes, and impedance [42,43,44]. In contrast, the Rudolph coefficient measures the relative timing and coordination between antagonist muscles [45,46]. Based on these observations, we hypothesized that EES drives stable temporal locomotor patterns despite raw signal power fluctuations. It should be emphasized that the electrophysiological changes observed under EES as reduced Rudolph coefficients and altered phase-plot patterns reflecting antagonist coordination in selected recordings do not correspond to a change in clinical neurological status. AIS grade remained B and LEMS remained zero throughout the 1-year follow-up, and these EMG findings should not be interpreted as evidence of AIS conversion, broad motor recovery, independent standing, or restored ambulation. The small changes observed at one year (the shift in neurological level from T3 to T4 and the 2- and 3-point increases in light-touch and pin-prick totals) fall within the range of measurement variability reported for the sensory component of the ISNCSCI exam in chronic SCI, particularly at the level of individual dermatomes and neurological level determination [47], and should therefore be interpreted with caution. Future prospective cohort studies remain necessary to validate whether this stability stems from permanent spinal circuitry reorganization.

Rhythmic reciprocal activation of antagonist muscles in response to EES-enabled motor activity in patients with SCI was demonstrated previously [1,6,39]. This study’s comparative analysis revealed distinct differences in EMG activity between trial Perc-EES and Paddle-EES (1y) one year after implantation. Notably, the participant received no specialized in-clinic rehabilitation during the 1-year follow-up. Training consisted of a structured, prescribed task-specific home-based program performed with stimulation assistance, with session duration progressively extended over time. These sessions were conducted under the remote video supervision of the research physician and directly utilized pre-programmed, task-specific EES configurations explicitly tailored to elicit and facilitate targeted leg movements. Paddle-EES (1y) showed more consistent and robust activation patterns following one year of home-based EES-facilitated training. Although previous research had shown the potential for some modulation of volitional movement even without specialized clinical rehabilitation [26], isolating the independent contribution of chronic epidural stimulation from the effects of regular home-based training sessions remains challenging. Accordingly, chronic stimulation, rehabilitation exposure, motor learning, and possible activity-dependent plasticity can be considered as interacting contributors. We hypothesize that these factors interact synergistically, as the stimulation modulates the excitability of residual spinal networks while task-specific home exercises help stabilize alternating muscle activation patterns. Optimizing these targeted physical rehabilitation strategies is critical to mitigate long-term disability from complex spinal disorders [48], especially given the substantial public health and socio-economic burden of neurological disabilities [49]. Therefore, refining patient selection and individual-level stimulation protocols was essential not only for advancing this single case but for the broader goal of improving functional outcomes in patients with SCI, thereby helping to reduce the clinical and economic burden associated with this condition. Without long-term training, attempts at isolated volitional movements typically yield minimal, restricted EMG responses, often masked by widespread co-contraction of opposing muscle groups [39]. Our study supported these findings and might further demonstrate the feasibility of using Perc-EES for early assessment. Although the focus of this study was the modulation of volitional movements in lower extremities, we also found improvement in autonomic functions such as control of micturition during trial and then chronic EES, similar to early studies showing that EES at L4-S1 restores voiding in humans with SCI [8,50].

Current studies on EES consists primarily of isolated case reports and small case series. As noted by Chalif et al. (2024), this limits the availability of standardized candidate selection criteria and leads to variability in patient outcomes and stimulation protocols [51]. As this study was also a single-case report based on limited evidence, these observations should be viewed as exploring merely one possible approach to patient selection and optimization rather than establishing definitive clinical guidelines. A key research opportunity lies in identifying which clinical profiles benefit most from EES. It remains unclear whether EES is equally effective for all chronic SCI patients or if it primarily benefits specific subpopulations, such as those with anatomically incomplete injuries. Personalizing neuromodulation protocols based on the ASIA classification—particularly for AIS A and B subjects—is an important area of study. Although studies [1,12,19,26] show the efficacy of EES in motor-complete SCI, baseline clinical classifications and neuroimaging are often insufficient to predict functional success. This is due to the microstructural heterogeneity of chronic lesions and variations in individual anatomy and residual supraspinal connectivity [9,52], meaning that the functional gains achieved by our single participant may reflect a highly specific neuroplastic potential.

Methodologically, the outcome of this case study provided important insights by directly comparing the effects of Perc-EES (two 8-lead rod electrodes) and Paddle-EES (16-lead paddle array). Implantation of two rod electrodes is commonly used for pain treatment in cases when bilateral pain cannot be well controlled with a single lead. Two-lead trial configuration in this study helped to increase the number of possible stimulation configurations during the trial period and compare with configurations used for Paddle-EES. Recent studies also demonstrated that the effect of EES may correlate with the segment-specific spinal cord anatomy, emphasizing the role of electrodes positioning and configurations opening new directions of anatomy-specific spinal cord neuromodulation [7,26,31,53,54,55,56,57,58,59,60,61]. By providing immediate feedback on the EES effects, trial stimulation can help to inform optimal neuromodulation and rehabilitation strategy and adjust subsequent Paddle-EES implantation to maximize therapeutic outcomes. Given the current lack of selection consensus, temporary percutaneous trial electrodes could be considered as a practical functional screening strategy. This approach is generally considered a relatively low-risk, reversible method that may help to assess neural circuitry responsiveness and to evaluate candidate eligibility before permanent implantation. Finally, clinical outcomes depend on addressing surgical and post-operative challenges. Standardizing the intervention requires precise surgical planning, electrode positioning, and intraoperative neurophysiological monitoring [53]. After implantation, clinical translation can be slowed by the technical complexities of programming multi-channel parameters like frequency, pulse width, and amplitude [62]. Additionally, achieving functional gains typically requires long-term rehabilitation, which may challenge standard clinical resources and contribute to variability in patient outcomes. Future research should focus on prospective cohorts, standardized stimulation and training protocols, appropriate controls, and predefined physiological and functional outcomes to validate these findings and explore the long-term benefits of combining trial Perc-EES and Paddle-EES with comprehensive rehabilitation–neuromodulation programs. Investigating the mechanisms underlying the rapid plasticity observed with Perc-EES could further provide deeper insights into optimizing neuromodulation therapies for SCI and other neurological disorders.

## 5. Conclusions

This study demonstrates the preliminary clinical feasibility of trial epidural electrical stimulation as a tool for pre-surgical neurophysiological evaluation in patients with motor-complete SCI (AIS B). Both trial Perc-EES and Paddle-EES facilitated comparable volitional motor activity below the injury level in supine, side-lying, and upright positions. These findings suggest that temporary stimulation may help assess acute neuromodulatory effects, optimize electrode placement, and guide individual rehabilitation strategies. This experience provides an initial framework for evaluating neurophysiological responsiveness prior to permanent implantation, although its broader clinical utility remains to be validated in larger cohorts. Although this study primarily focused on volitional movements in the lower extremities, case-specific improvements in autonomic functions were also observed, including micturition control during both trial and chronic phases and enhanced colorectal regulation during chronic stimulation. These preliminary findings are hypothesis-generating and suggest that EES may modulate neural circuits involved in autonomic control, although further research is needed to evaluate any generalized therapeutic effect on visceral functions in humans with SCI.

## 6. Limitations

Several limitations must be acknowledged: First, due to the single-patient design (*n* = 1), these preliminary findings could not be directly generalized to a broader population, and the observed distinct changes reflected within-subject variance rather than population-level data, serving primarily as a proof of hypothesis. Second, the participant’s specific clinical profile—a chronic (>20 years), motor-complete (AIS B) injury at Th5–Th6 of non-traumatic mixed etiology—means that individual anatomy, lesion topography, and neuroplastic potential might differ from more common traumatic SCI. Thus the results of the study should not be extrapolated to broader SCI population without prospective replication. Furthermore, procedural factors limited direct comparisons between the study phases. The use of different epidural leads during the trial (two 8-contact percutaneous Abbott leads) and chronic phases (5-6-5 Medtronic paddle lead) altered current distribution and motor thresholds due to variations in length of coverage, contact position, and spacing. The selection of EMG-recording intervals for analysis was not blinded, which can cause potential bias. Additionally, because the structured home-based rehabilitation tasks were designed around the stimulation, the individual contributions of EES and motor training could not be fully isolated; the outcomes must be interpreted as a combined effect. Adherence to the home program was documented via weekly self-reports and monthly telehealth consultations without wearable-based activity monitoring. Future large-scale, prospective trials incorporating objective tracking, such as accelerometry, are strictly required to validate this prognostic tool and evaluate its broader clinical applicability. Finally, the study was not deposited in a public registry which limits external verifiability of the design and outcome definitions, and readers should interpret the findings with this consideration in mind.

## Data Availability

The raw data are available upon reasonable request.

## Supplementary Materials

The following supporting information can be downloaded at https://www.mdpi.com/article/10.3390/jcm15176663/s1, Table S1: Examination and neurological assessment; Table S2: Results of laboratory and instrumental examination before enrollment; Table S3: EMG amplitudes (mV) of lower limb muscles during the flexion–extension test; Table S4: Rudolph coefficient values in vertical and side-lying positions; Table S5. Summary of Clinical and Electrophysiological Outcomes Across Study Phases; Figure S1: Spinally evoked motor potentials (SEMPs) during trial stimulation and stimulation with paddle electrode arrays. (A) Trial epidural stimulation with different electrode configurations induced SEMPs on the left and right legs. Intraoperative recordings of SEMPs were collected from proximal (RF, rectus femoris; BF, biceps femoris; VL, vastus lateralis) and distal (GM, medial gastrocnemius; TA, tibialis anterior; SOL, soleus) muscles. (B) Paddle-EES with electrode configuration at the midline (5+10-). (C) The amplitudes of the EES evoked motor responses of proximal and distal muscles during left- and right-side Perc-EES, and with midline stimulation with Paddle-EES (*  ≤ 0.05; #  ≤ 0.001). Note that Y-axis scales are identical for all amplitude bar charts in Panel C, whereas the scales for representative curves are individualized and should not be used for direct amplitude comparison; Figure S2: Area under the curve (AUC) during Perc- and paddle electrode arrays (*  ≤ 0.05; #  ≤ 0.001). Multiple statistical comparison brackets are retained across muscle and position combinations to demonstrate the absence of any uniform or unidirectional dynamics in the EMG area under the curve (AUC); Supplementary Video S1. Effect of EES on voluntary motor activity of the leg muscles. (A) Example of voluntary motor activity without and with Perc-EES (trial EES). (B) Example of EES-enabled voluntary motor activity with Perc-EES (trial EES) (after motor training with biofeedback). (C) Example of EES-enabled voluntary motor activity after one year of EES-enabled training; Supplementary Video S2. Example of voluntary step-like activity in side-lying position during Perc-EES and after one year of EES-enabled training with Paddle-EES (1y); Supplementary Video S3. Example of rhythmic movements in upright position without and with EES. File S1. Materials and Methods [63,64,65,66].

## Abbreviations

The following abbreviations are used in this manuscript:

BWS	Body weight support
EES	Epidural electrical stimulation
Paddle-EES	Chronic paddle-electrodes epidural electrical stimulation
Perc-EES	Percutaneous-epidural electrical stimulation
SCI	Spinal cord injury
AIS	American Spinal Injury Association Impairment Scale
CSF	Cerebrospinal fluid
UTI	Urinary tract infection
MRI	Magnetic resonance imaging
EMG	Electromyography
AUC	Area under the curve
SEMP	Spinally evoked motor potential
BF	Biceps femoris
RF	Rectus femoris
TA	Tibialis anterior
GM	Medial gastrocnemius
VL	Vastus lateralis
SOL	Soleus
AIS	ASIA Impairment Scale
NLI	Neurological level of injury
LEMS	Lower extremity motor score
VAC	Voluntary anal contraction
DAP	Deep anal pressure
