# Supplementary material for: From Trial to Chronic Epidural Electrical Stimulation: A Single-Patient Feasibility Study in Motor-Complete Spinal Cord Injury
Source: J Clin Med. 2026 Aug 28;15(17):6663. doi: 10.3390/jcm15176663 (PMC13566598; doi:10.3390/jcm15176663)
Supplement: Supplementary file 1 [file jcm-15-06663-s001.zip › File S1 Materials and Methods.pdf]

Materials and Methods

Examination

Prior to study enrollment, routine laboratory tests, including complete blood count, blood chemistry test, and urinalysis, as well as instrumental examinations such as MRI and densitometry, were performed (Supplementary Tables S1, S2). To assess motor and sensory impairment and the severity of spinal cord injury, the International Standards for Neurological Classification of Spinal Cord Injury (ISNCSCI), commonly referred to as the American Spinal Injury Association (ASIA) Impairment Scale (AIS), were used. In addition, questionnaires were used to evaluate quality of life, mental health, and bowel, bladder, and sexual function. One day before trial electrode implantation, EMG activity was recorded during attempts of voluntary movements. The participant was instructed to perform voluntary flexion and extension in three joints (ankle, knee, and hip) bilaterally, followed by general flexion of the hips and knees of both legs. All motor tasks were performed in a supine position, both without and with the Jendrassik reinforcement maneuver.

Neurological examination

Detailed physical examination findings are provided in Supplementary Table S1 and included the following: a) Limb Strength: normal in arms bilaterally; 0/5 in legs. b) Sensory: loss of sensation below T3 with partial sensation at T4 and T5 levels; total loss of sensation below Th6; positive DAP (deep anal pressure); negative VAC (voluntary anal contraction); no movements below the trauma level. c) Reflexes: Absent bulbocavernous reflex, decreased Deep Tendon Reflexes in bilateral knees and ankle joints. d) Muscle Tone: Normal in upper extremities; 1+ in lower extremities (Ashworth scale). e) Pathological Reflexes: Positive Babinski sign bilaterally. f) Bowel and Bladder Function: Voiding difficulty. g) Cognition: MoCA (Montreal Cognitive Assessment) score of 28 out of 30.

Study design and timeline

After enrollment and initial evaluation, the participant was implanted with two trial 8-contacts electrodes (Multiprogram Trial Stimulator (MTS), 3510, Abbott, Plano, TX, USA) at the lumbosacral segments with the subsequent testing started 2 days after implantation (Figure1B). After trial stimulation performed for one week, the participant was reimplanted with a chronic 5-6-5 Medtronic array (Medtronic, Fridley, MN, USA) with the subsequent testing started 2 days after implantation. Positioning of the trial and chronic electrodes within the T11–L1 vertebrae corresponding to L1–S1 spinal segments [31,53,63] was performed using anatomical landmarks and fluoroscopy (Figure 1C). To confirm symmetric position, leads on both trial and chronic electrode arrays were used to evoke and compare motor response on the left and right leg muscles (Supplementary Figure S1). Test consequence (supine, side-lying, vertical position) was dictated by two factors: (1) to avoid postoperative complications after EES implantation caused by early applied stimulation and (2) to prepare the participant for gradual adaptation to the complexity of the functional task.

Implantation for percutaneous trial EES

The trial electrode implantation procedure was performed under local anesthesia with the participant in a prone position. Following sterile preparations, the posterior epidural space was punctured with a Tuohy needle at the L3-L4 level bilaterally adjacent to the spinous process. Two

eight-contact leads (Octrode, model 3086; Abbott, Plano, TX, USA, 3 mm lead with 4 mm spacing) were placed over the dorsal epidural surface of the lumbosacral spinal cord bilaterally from the caudal edge of the Th11 vertebra to the middle part of L1, covering the L2-S1 spinal cord segments. The Multiprogram Trial Stimulator (MTS), model 3510, Abbott, Plano, TX, USA, was used for stimulation. The contacts A1-8 corresponded to the left of the central line (left lead), while contacts B1-8 corresponded to the right side (right lead), with a distance of ~16.7 mm between the right and left arrays at the proximal contacts and ~11.0 mm at the distal contacts (Figure 1C). Previous reports indicate that electrode leads placed in the projection of the T11-L1 vertebrae correspond to the L1–S1 spinal segments [31,38,39]. The trial electrodes were positioned from the caudal edge of the Th11 vertebra to the middle of L1 (Figure 1C).

Implantation for chronic paddle lead EES

Chronic electrode implantation was performed under general anesthesia with the participant in a prone position with a soft roller under the abdomen to expand interspinous space. Prior to midline incision fluoroscopy marking was performed and L2-L3 vertebrae were exposed using standard surgical techniques. Partial laminectomy of the L2-L3 vertebrae was made by 16-contact epidural electrode array (Specify 5-6-5; Medtronic, Fridley, MN, USA) are placed positioned on the dorsal epidural surface of the lumbosacral spinal cord (electrode was placed in the space from the rostral edge of Th12 vertebra to the middle of L1 including L2-L5, S1 under fluoroscopy control and electrophysiological testing to precise position. The lead was fixed with clamps to the fascia. Then the pulse generator was implanted in a pocket in the right lumbar region without changing the participant's position on the operating table.

Intraoperative electrophysiological assessment

Immediately after trial electrode implantation, impedance was measured for all contacts and was found to be below 1000 Ω. The location of the electrode array was evaluated during surgery by recording the thresholds and amplitudes of spinally evoked motor potentials (SEMP) from proximal and distal muscles of the legs, elicited by stimulation at 250 μs, 2 Hz. Two 8-contact electrode arrays (3 mm lead with 4 mm spacing, Abbott, Plano, TX, USA) were placed in the epidural space from the caudal edge of the Th11 vertebra to the middle part of L1, covering spinal cord from L2 to S3 spinal cord segments as described above (Figure 1C). During chronic stimulation, a 16-contact paddle electrode array (Medtronic 5-6-5, Medtronic, Fridley, MN, USA) was used. Symmetrical position of the electrodes was tested using midline, left, and right-side contacts within the array (trial system: wide field configurations (1+8-); chronic system: wide field configurations (5+10-)). Supplementary Figure S1 provides examples of SEMPs evoked by the lowest EES intensity, involving the major muscles of the lower limbs.

Electrophysiology

Electrophysiological assessment was performed prior to surgery, intraoperatively, and during postoperative testing. EMG record settings were consistent for all registrations. EMG was recorded from the following muscles: rectus femoris (RF), vastus lateralis (VL), biceps femoris (BF), tibialis anterior (TA), medial gastrocnemius (GM) and soleus (SOL) muscles bilaterally. For pre-and post-operative testing, surface electrodes were used, for intraoperative testing 13-mm subdermal electrodes were placed over selected muscles of the lower limbs. Prior to electrode placement, the skin

was prepared by hair removal and cleaning with alcohol wipe. The inter-electrode distance was standardized at 2 cm for all recordings, in accordance with standard electrophysiological recording The electrodes were applied over the midpoint of the appropriate muscle. A ground electrode was placed on the anterior thigh. During intraoperative testing additional two electrodes were placed over paraspinal muscles bilaterally to record a stimulation artefact, to sweep the trigger. During preoperative EMG testing the participant lied supine with head elevation enough for observing legs movements. A three-minute relaxation was performed to differentiate involuntary (spasticity) and volitional movements.

EES-enabled motor training and EES-enabled motor assessment: After the implantation of both trial and chronic systems, EES-enabled volitional motor control was assessed. Two types of tests were performed: a flexion-extension test to evaluate the ability to produce volitional activation in multiple joints (progressing to isolated muscle activation of a single joint) and rhythmic movements at a self-selected speed in both side-lying and upright positions. Each session included three trials: before stimulation, during stimulation, and immediately after stimulation. Each motor task was repeated three times to identify the optimal EES electrode configurations and stimulation settings for enabling optimal volitional control with EES. The intensity of EES was gradually increased until volitional control over leg muscles was observed.

EES-enabled home-based motor training

Following parameter optimization, the participant performed home-based stimulation assisted motor training combined with stimulation, comprising 6 sessions per week: 2 side-position trainings with one leg suspended, 2 sitting-position trainings, and 2 vertical trainings using a walking frame for support.

Side-position training was performed with contacts 3+/4− and 14+/15−, pulse width 250 mks, frequency 20 Hz, and amplitude limits 6.5–7.5 V. The initial session duration was 30 minutes (15 minutes per leg); every three months, the duration was increased by 5 minutes per leg every three months from month 3 onwards, reaching 70 minutes per session by the 12-month follow-up. Within a system in which the patient activated cyclical leg movements using stimulation, the patient attempted to control the initiation and cessation of movement, to increase the amplitude and speed of the cyclical pattern, and to perform isolated joint movements (individually at the ankle, knee, or hip). He also attempted to flex one leg while simultaneously extending the other.

Sitting-position (balance) training was performed with contacts 0+/2− and 4+/13−, pulse width 250 mks, frequency 20 Hz, and amplitude limit 9 V. The initial session duration was 30 minutes and was subsequently increased by 5 minutes each month, reaching 1.5 hours by the follow-up examination at one year. The patient attempted to sit on a level surface with the legs extended, maintaining a maximally upright trunk, initially with hand support. He subsequently progressed to sitting without hand support, with the arms extended forward, upward, and laterally. Further tasks included reaching as far forward as possible and returning to the midline without hand support, and reaching laterally with the arms extended, followed by a return to the central position. Over the course of the year, both the session duration and the number of attempts were progressively increased.

Vertical training with a walker was performed with contacts 0+/4− and 11+/15−, pulse width 250 mks, frequency 20 Hz, and amplitude limits 6.5–7 V. The initial session duration was 30 minutes and was subsequently increased by 5 minutes each month, reaching 1.5 hours by the follow-up examination at one year. Training focused on maintaining upright balance, weight shifting from one leg to the other, active maintenance of knee extension during weight-bearing, and attempting stepping movements with stimulation.

Stimulation parameters were largely stable throughout follow-up; minor amplitude adjustments were made at monthly review when required to maintain motor threshold, without changes to contact configuration, pulse width, or frequency."

All home training was performed without of any assistance throughout the session.

Remote clinical monitoring consisted of weekly reports, in which the participant submitted data logs on training duration and EES parameters to the investigator, together with monthly telehealth video sessions with the physician to evaluate movement quality and adjust the training protocol. Over the course of the home rehabilitation period, the participant reported an increase in physical activity tolerance. During this time, no significant health issues, including no autonomic dysreflexia, no device- or wound-related complications, no worsening of spasticity requiring medical treatment or affecting daily activity, were observed, including no side effects of stimulation requiring treatment or affecting everyday activity.

Data Processing and Analysis

EMG data was recorded using LabChart software and Bioamp, PowerLab system (ADInstruments, Inc., Dunedin, New Zealand), filtered using a 50-Hz notch filter and a bandpass filter of 20 to 1000 Hz, and sampled at 4 kHz. All data were then exported and analyzed using MATLAB software (The MathWorks, Inc., Natick, MA, USA) and a custom Delphi algorithm (Embarcadero Inc., Austin, TX, USA).

Intraoperative Monitoring: EMG data collected during intraoperative monitoring was analyzed as follows: ten evoked responses were averaged for each stimulation trial. The latency time of the spinally evoked motor potentials (SEMP) was defined as the time between stimulation onset and the evoked response onset. The SEMP amplitude was quantified by the peak-to-peak amplitude, measured in an interval calculated during the 50 ms stimulation after artifact removal using a MATLAB script. Evoked responses recorded during intraoperative monitoring were analyzed separately for each electrode configuration.

Voluntary Movement Activity: For the assessment of voluntary movement activity, the EMG signal was recorded for 5 seconds in the rest position prior to the movement and for 10 seconds during the execution of the indicated movement according to protocol. A background/resting threshold was calculated by extracting the first 5 seconds of data from each trial [64]. The unrectified EMG signal was windowed every 10 ms, and the difference between the minimum and maximum values in the window was calculated to produce a peak-to-peak value. The average value of these rest data was taken to establish the threshold for detecting motor activity for each channel. For quality control, a reanalysis with half a shift in windows was used. The number of detected motor units arising either during the command to move or at rest was calculated. The EMG was processed with the custom Delphi

algorithm to analyze peak-to-peak amplitude (Embarcadero Inc., Austin, Texas, USA). The EMG-response threshold was set at 0.05mV to remove background noise.

Rhythmic EMG Activity: For the analysis of rhythmic muscle activity, a 20-second interval was selected from a 3-minute recording. The selection was based on period of stable, continuous stepping. Between 10-20 patterns of EMG activity were collected for each muscle. The area under the curve (AUC) in the EMG signal of each muscle was calculated from the beginning of each pattern. To determine muscle activity coordination, plots of the dependence function of the average envelope of the EMG signals from the biceps femoris (BF) and vastus lateralis (VL) for proximal muscles, and tibialis anterior (TA) and soleus (SOL) for distal muscles, were generated over a selected time interval of more than 20 seconds with at least 10 selected bursts of EES-induced muscle activity. The characteristic shape of a curve for asynchronous EMG signals of two muscles is L-shaped, indicating minimum level of the envelope of the EMG signal of one muscle with the maximum level of the EMG signal of the other muscle. Graphs of the average envelope function of the EMG signals were calculated from the root mean square (RMS) value of the EMG activity, averaged over a window length of 200 samples at a data acquisition frequency of 4 kHz. The asynchrony of EMG signals from muscle pairs was quantified by calculating the Rudolph coefficient wich represents the co-contraction index proposed by Rudolph.The Rudolph co-contraction index represents the ratio of the amplitude of less active muscle (min (A_i, B_i)) to the amplitude of more active muscle (max (A_i, B_i)) at each time point *i* during the examined period multiplied by the sum of both amplitudes (A_i+B_i) at point *i*: $(A_i+B_i)\times(\min(A_i,B_i) / \max(A_i,B_i))$ [65]. Originally the Rudolph co-contraction index was used for caculating the cocontraction during hopping in patients with an anterior cruciate ligament rupture, but a number or reseachers used it to detect the coactivation during gate in post-stroce individuals, muscle coactivation in cerebellar ataxia patients and diring the transcutenious spinal cord stimulation in healthy individuals [45,66] Due to Rudolph et al., 2020 a high value of cocontrucrtion index represents a high level of activation of both muscles across an examined time interval while the low values indicate a high level activation of one muscle along with low level activation of the other muscle in the pair [65]. The Rudolph coefficient in our research is the averaged Rudolph co-contraction index divided in half. In this case the values of the coefficient approached to 'zero' showed asynchronous EMG signals and values that were close to 'one' showed synchronous EMG signals. Thus, during the reciprocal activity of antagonist muscles the Rudolph coefficient approaches to zero, while during their coactivation it will be close to one.

Statistical Analysis

Statistical Analysis processing was performed in OriginPro 2021 (OriginLab Corp., Northampton, MA, USA). Data normality and variance homogeneity were assessed using the Shapiro–Wilk and Levene's tests, respectively. Absolute, non-normalized values were used for all EMG metrics. The experimental design consisted of a single session per condition, evaluating three activation states (Perc-EES, Paddle-EES, and 1-year follow-up) across three experimental conditions (voluntary flexion-extension, side-lying rhythmic activity, and upright rhythmic activity). For the voluntary flexion-extension test, analysis was performed on a 10-s movement window

following a 5-s resting baseline, utilizing a pool of overlapping 50-ms sliding window amplitude estimates as the unit of analysis. For EES-facilitated rhythmic activity, a 20-s stable interval was extracted from a 3-min recording; the consecutive individual step cycles (up to 10) were isolating within each trial and handled by as independent units of analysis, the AUC calculated from pattern onset. For EES-facilitated rhythmic activity, a 20-s stable interval was extracted from a 3-min recording; repeated observations within each trial were handled by isolating up to 10 consecutive individual step cycles as independent units of analysis, with the AUC calculated from pattern onset. To assess inter-muscular coordination, Rudolph coefficients were calculated on a cycle-by-cycle basis, utilizing each isolated step cycle within the stable interval as the independent unit of analysis. Normally distributed data with equal variances were compared using a two-sample t-test for two groups or one-way ANOVA for multiple groups. Non-normally distributed data were analyzed using the Mann–Whitney U test or the Kruskal–Wallis test followed by Dunn's post-hoc pairwise comparisons. Data summaries express AUC, voluntary EMG amplitude, and spinally evoked motor potentials (SEMP) as mean ± standard error of the mean (SEM), representing the central tendency and dispersion of the respective window or cycle pools. Rudolph coefficients are reported as median and interquartile range (Q1–Q3). Statistical significance was set at $p<0.05$. All reported p-values reflect intraindividual changes at the registration level and do not imply population-level clinical efficacy.
