# Supplementary material for: From Trial to Chronic Epidural Electrical Stimulation: A Single-Patient Feasibility Study in Motor-Complete Spinal Cord Injury
Source: J Clin Med. 2026 Aug 28;15(17):6663. doi: 10.3390/jcm15176663 (PMC13566598; doi:10.3390/jcm15176663)
Supplement: Supplementary file 1 [file jcm-15-06663-s001.zip › Table S1.pdf]

Supplementary Table S1. Examination and neurological assessment

Scales				
		Time points		
		Before enrolment	~ 1m after chronic system EES implantation	~ 1y after chronic system EES implantation
ISNCSCI	NLI	T3	T3	T4
	AIS	B	B	B
	LER	0	0	0
	LEL	0	0	0
	LEMS total	0	0	0
	UER	25	25	25
	UEL	25	25	25
	UEMS total	50	50	50
	LTR	23	23	24
	LTL	22	22	23
	LT total	45	45	47
	PPR	23	23	24
	PPL	22	22	24
	PP total	45	45	48
	VAC	No	No	No
	DAP	Yes	Yes	Yes
	Pathological reflexes	+	+	+

	Spasm frequency (PSFS)	3	0	1
	Pain (VAS)	NA	NA	NA
	Pain management	no	no	no
	Rehabilitation	+	+	+
	Level of lesion	Th5-6	Th5-6	Th5-6
	Length of lesion	Th5-6	Th5-6	Th5-6
	Blood presence	no	no	no
ASAF	AC of the heart	N	N	N
	AC of the BP	N	N	N
	AC of sweating	N	N	N
	Temperature regulation	N	N	N
	AC and SC of BPS	N	N	N
	Bladder management	3	3	3
	Bowel management	3	3	3
	Sexual function	3	3	3
	BP at rest (mean)	>110/70 and <130/90	>110/70 and <130/90	>110/70 and <130/90
	HR at rest	67-85	72- 87	65 - 85
	Orthostatic test (lying down – sit)	NEG	NEG	NEG
	History of AD	no	no	no
	Medications	no	no	no

SCIM	Self-Care (out of 20)	18	18	18
	Respiration and sphincter management (out of 40)	39	39	39
	Mobility (out of 40)	19	19	19
	Total (out of 100)	76	76	76
NBDS	Total	6	3	3
	GS (out of 10)	8	10	9
NBSS	Incontinence (out of 29)	8	7	7
	Storage/voiding (out of 22)	12	9	8
	Consequence (out of 23)	5	5	2
	QOL (out of 4)	2	2	1
	Management method	D	D	D
PHQ9		3	3	0
SF – 36	Physical functioning	20	15	25
	Role limitations due to physical health	25	25	50
	Role limitations due to emotional problems	100	100	100
	Energy/fatigue	70	70	70
	Emotional well-being	72	72	72
	Social functioning	100	75	100

	Pain:	80	80	80
	General health:	50	50	50
	Health change:	50	75	50
MAS		1+	0	1

ISNCSCI – International Standard for neurological classification of spinal cord injury
NLI – neurological level of lesion
AIS – ASIA (American spinal cords injury association) Impairment Scale
LEMS – lower extremities motor subscores
UEMS – upper extremities motor subscores
VAC – voluntary anal sphincter contraction
DAP – deep anal pressure
Pathological reflexes
PSFS – Penn Spasm Frequency Scale
VAS – visual analog scale
ASAF – Autonomic Standard Assessment Form
AC – autonomic control
BP – blood pressure
BPS – broncho-pulmonary system
SC – Somatic control
HR – heart rate
HBL – hyperhidrosis below lesion
SF-Spasm frequency
OT – orthostatic test
History of AH – arterial hypertension
SCIM III – Spinal Cord Independence Measure
NBDS – The Neurogenic Bowel Dysfunction Score (GS-general satisfaction)
NBSS – The neurogenic Bladder Symptom Score (QOL – quality of life)
PHQ9 – Patient Health Questionnaire-9
SF – 36 – The Short Form-36
MAS – Modified Ashworth Scale
NEG -negative
N- normal
