# Supplementary material for: From Trial to Chronic Epidural Electrical Stimulation: A Single-Patient Feasibility Study in Motor-Complete Spinal Cord Injury
Source: J Clin Med. 2026 Aug 28;15(17):6663. doi: 10.3390/jcm15176663 (PMC13566598; doi:10.3390/jcm15176663)
Supplement: Supplementary file 1 [file jcm-15-06663-s001.zip › Table S2.pdf]

Supplementary Table S2

Blood component		
	Participant’s value	Reference range
White blood cells (WBC)	6.2 x 109/L	4.5-11.0 x 109/L
Red blood cells (RBC)	4,9 x 1012/L	4.3-5.9 x 1012/L
Hemoglobin (HGB)	160 g/L	135-175 g/L
Hematocrit (HT)	0,44	0.41-0.53
Mean corpuscular volume (MCV)	90,4 fl	80-100 fl
Mean corpuscular hemoglobin (MCH)	31.6 pg	28-32 pg
Mean corpuscular hemoglobin concentration (MCHC)	35	30-38 g/dL
Platelets	213 x 109/L	150-400 x 109/L
Biochemical blood test		
	Participant’s value	Reference range
Transaminases: ALT	25.5669 U / L	Up to 37 U / L
Transaminases: ACT	22.4586 U / L	Up to 45 U / L
Total protein	69.7418 g / l	60 - 85 g / l
Chol (cholesterol)	5.6853	2.2 - 5.7 mmol / liter

Sugar (glucose)	5.1443 mmol / l	3.8 - 6.3 mmol / l
Urea	6.8004 mmol / l	2.8 - 7.2 mmol / l
Total bilirubin (tbil, bil)	10.019	8.5 - 20.5 µmol / l
Fibrinogen		2 - 4 g / l
Creatinine	93.122 µmol / l	62 - 120 µmol / l
Lipoprotein HDL	1.24 mmol / l	0.9 - 1.8 mmol / l
Lipoproteins of LDL	3.8 mmol / l	1.8 - 4.9 mmol / l
Amylase (amyl)	76 U/L	25 - 125 U/L
Triglycerides	1.2698	0.55 - 2.26 mmol / l
Uric acid	336,1638	210 - 420 µmol /L
Urinalysis		
Color	Bright yellow	
Concentration.	1015	
Protein.	neg	
Sugar	neg	
WBC	1-2	
RBC	0	
Epitelial cells	0-1	
Instrumental evaluation		
Thoracic spine MRI (20/06/2022)	Condition after surgical treatment (14.05.2001.19.07.2016) at the level of the Th5-Th6 segment of the pattern of weak	

	diffuse accumulation of contrast in the area of postoperative changes in the spinal cord, without signs of progressive growth. Compared with the MRI study of 12.07.2017 without any signs of dynamics. Atrophic changes in the spinal cord. Hemangiomas of the bodies of Th8, Th11 vertebrae.
Bone mineral density	At lumbar spine (T-criterion -0.1 SD; Z-criterion -0.2 SD), at the left femoral bone (T-criterion -2.7 SD; Z-criterion -2.5 SD), at the right femoral bone (T-criterion -2.8 SD; Z-criterion -2.7 SD).
