# Supplementary material for: From Trial to Chronic Epidural Electrical Stimulation: A Single-Patient Feasibility Study in Motor-Complete Spinal Cord Injury
Source: J Clin Med. 2026 Aug 28;15(17):6663. doi: 10.3390/jcm15176663 (PMC13566598; doi:10.3390/jcm15176663)
Supplement: Supplementary file 1 [file jcm-15-06663-s001.zip › Table S3.pdf]

Table S3. EMG Amplitudes (mV) of Lower Limb Muscles During the Flexion-Extension Test.

Movement & Limb	Muscle	No Stim, mV	Perc-EES, mV	Paddle-EES, mV	P-value
**Right flexion – Left extension**					
Right Leg (Flexion)	RF	0	0	0	N/A
	BF	0	0.110 ± 0.002	0.062 ± 0.002	Perc-EES vs. Paddle-EES: < 0.001; No Stim: N/A
	VL	0	0	0.061 ± 0.005	N/A
	TA	0	0	0.165 ± 0.012	N/A
	GM	0	0	0	N/A
	SOL	0	0	0.074 ± 0.002	N/A
Left Leg (Extension)	Left TA	0.086 ± 0.005	0	0	N/A
	RF	0	0	0.074 ± 0.003	N/A
	BF	0	0.119 ± 0.001	0.067 ± 0.003	Perc-EES vs. Paddle-EES: < 0.01; No Stim: N/A
	VL	0	0.196 ± 0.002	0.076 ± 0.003	Perc-EES vs. Paddle-EES: < 0.01; No Stim: N/A
	TA	0	0.063 ± 0.003	0.255 ± 0.008	Perc-EES vs. Paddle-EES: < 0.01; No Stim: N/A
	GM	0	0.076 ± 0.004	0.059 ± 0.003	Perc-EES vs. Paddle-EES: < 0.01; No Stim: N/A
	SOL	0	0.065 ± 0.004	0.065 ± 0.002	Perc-EES vs. Paddle-EES: NS; No Stim: N/A
**Right extension – Left flexion**					
Right Leg (Extension)	RF	0	0	0	N/A
	BF	0	0	0	N/A
	VL	0	0	0	N/A
	TA	0	0	0.086 ± 0.005	N/A
	GM	0	0	0	N/A
	SOL	0	0	0	N/A

Left Leg (Flexion)	RF	0	0	0.067 ± 0.004	N/A
	BF	0	0	0	N/A
	VL	0	0.069 ± 0.001	0.089 ± 0.005	Perc-EES vs. Paddle-EES: < 0.001; No Stim: N/A
	TA	0.348 ± 0.008	0.650 ± 0.009	0.902 ± 0.060	Perc-EES vs. Paddle-EES: < 0.01; No Stim: < 0.001
	GM	0	0.074 ± 0.001	0.069 ± 0.003	Perc-EES vs. Paddle-EES: < 0.001; No Stim: N/A
	SOL	0	0.177 ± 0.002	0.089 ± 0.004	Perc-EES vs. Paddle-EES: < 0.001; No Stim: N/A

Note: Values for No Stim, Perc-EES, and Paddle-EES are presented as Mean ± SE. "NS" denotes non-significant statistical differences (P ≥ 0.05). "N/A" indicates instances where no statistical test was performed due to the lack of physiological recruitment.
