# Supplementary material for: From Trial to Chronic Epidural Electrical Stimulation: A Single-Patient Feasibility Study in Motor-Complete Spinal Cord Injury
Source: J Clin Med. 2026 Aug 28;15(17):6663. doi: 10.3390/jcm15176663 (PMC13566598; doi:10.3390/jcm15176663)
Supplement: Supplementary file 1 [file jcm-15-06663-s001.zip › Table S4.pdf]

Table S4. Rudolph Coefficient Values in Vertical and Side-Lying Positions.

Body Position	Muscles (Antagonists)	Perc-EES	Paddle-EES	Paddle-EES (1y)	Statistical Significance (*P*-value)
**Upright**	RF vs BF L	0.29 [0.19; 0.44] (n=8)	0.39 [0.29; 0.54] (n=10)	0.09 [0.02; 0.33] (n=9)	Perc-EES vs Paddle-EES: **0.07**; Perc-EES vs Paddle-EES (1y): **<0.001***; Paddle-EES vs Paddle-EES (1y): **0.001***
	RF vs BF R	0.615 [0.51; 0.69] (n=8)	0.585 [0.5; 0.66] (n=10)	0.05 [0.02; 0.11] (n=9)	Perc-EES vs Paddle-EES: **0.264**; Perc-EES vs Paddle-EES (1y):**<0.001***; Paddle-EES vs Paddle-EES (1y): **<0.001***
	TA vs GM L	0.695 [0.12; 0.89] (n=8)	0.205 [0.08; 0.51] (n=10)	0.11 [0.04; 0.33] (n=9)	Perc-EES vs Paddle-EES: **0.177**; Perc-EES vs Paddle-EES (1y): **<0.001***; Paddle-EES vs Paddle-EES (1y): **<0.001***
	TA vs GM R	0.7 [0.63; 0.86] (n=8)	0.815 [0.4; 0.9] (n=10)	0.03 [0.01; 0.09] (n=9)	Perc-EES vs Paddle-EES: **0.687**; Perc-EES vs Paddle-EES (1y): **P<0.050**; Paddle-EES vs Paddle-EES (1y): **P<0.050**
**Side-Lying**	RF vs BF L	0.48 [0.31; 0.72] (n=9)	0.3 [0.06; 0.56] (n=8)	0.16 [0.01; 0.3] (n=9)	Perc-EES vs Paddle-EES:**0.017**; Perc-EES vs Paddle-EES (1y): **0.076**; Paddle-EES vs Paddle-EES (1y): **<0.001***
	RF vs BF R	0.44 [0.3; 0.59] (n=9)	0.29 [0.11; 0.47] (n=9)	0.095 [0.01; 0.3] (n=8)	Perc-EES vs Paddle-EES:**0.027**; Perc-EES vs Paddle-EES (1y): **0.004***; Paddle-EES vs Paddle-EES (1y): **<0.001***
	TA vs GM L	0.43 [0.3; 0.47] (n=9)	0.825 [0.66; 0.85] (n=8)	0.27 [0.01; 0.4] (n=9)	Perc-EES vs Paddle-EES:**<0.001***; Perc-EES vs Paddle-EES (1y): **0.001***; Paddle-EES vs Paddle-EES (1y): **<0.001***
	TA vs GM R	0.59 [0.33; 0.69] (n=9)	0.695 [0.59; 0.76] (n=8)	0.22 [0.1; 0.46] (n=5)	Perc-EES vs Paddle-EES: **0.018**; Perc-EES vs Paddle-EES (1y): **<0.001***; Paddle-EES vs Paddle-EES (1y): **<0.001***

Data represent the Rudolph coefficient (median [minimum; maximum] (n=steps)). Statistically significant differences (P≤0.05) are highlighted in bold; highly significant differences (P≤0.001) are marked with an asterisk *. Higher values indicate greater co-activation, while values closer to zero indicate optimal reciprocal coordination.
