# Supplementary material for: From Trial to Chronic Epidural Electrical Stimulation: A Single-Patient Feasibility Study in Motor-Complete Spinal Cord Injury
Source: J Clin Med. 2026 Aug 28;15(17):6663. doi: 10.3390/jcm15176663 (PMC13566598; doi:10.3390/jcm15176663)
Supplement: Supplementary file 1 [file jcm-15-06663-s001.zip › Table S5.pdf]

Table S5. Summary of Clinical and Electrophysiological Outcomes Across Study Phases

Functional Domain	No EES	Perc-EES	Paddle-EES	Paddle-EES (1 Year Post-Implant)
**Flexion-Extension Test**	No visible movements	Partial recruitment (BF only during flexion)	Full recruitment of all muscles (BF, VL, TA, SOL, RF, GM)	**Not performed**
**Rhythmic Activity (Side-Lying)**	No visible movements	Asynchronous signals and co-contraction	Improved proximal and distal muscle coordination	Enhanced coordination between muscles antagonists
**Upright Position**	No visible movements	Immediate restoration of volitional movements	Coordination patterns identical to Perc-EES phase	Substantial reduction of muscle co-activation
**Clinical Assessment**	• NLI: T3; AIS: B<br>• Sensory:<br>LT total: 45<br>PP total: 45<br>• LEMS total: 0 points<br>• NBSS: 8 / 12 / 5 / 2<br>• NBD / Bowel Satisfaction: 6 / 8	No changes except immediate restoration of voiding initiation during Perc-EES	• NBSS: 7 (Incontinence) / 9 (Storage) / 5 / 2<br>• NBD / Bowel Satisfaction: 3 / 10	• Sensory:<br>LT total: 45 → 47<br>PP total: 45 → 48<br>• NLI shifted: T3 → **T4**;<br>• NBSS: 7 / 8 / 2 / 1;<br>NBD / Satisfaction: 3 / 9

Note on Phase Diagrams: The evolution toward an L-shaped trajectory in the phase diagrams is a hallmark of functional reciprocal activation and Note on Assessment Timelines: The volitional "flexion-extension" test was not performed during the 1-year post-implantation assessment.Abbreviations: EES, epidural electrical stimulation; NLI, neurological level of injury; AIS, ASIA Impairment Scale; NBSS, Neurogenic Bladder Symptom Score; QoL, quality of life; NBD, Neurogenic Bowel Dysfunction; EMG, electromyography;AUC, area under the curve;BF, m. biceps femoris;VL, m. vastus lateralis;TA, m. tibialis anterior;SOL, m. soleus;RF, m. rectus femoris; GM, m. gastrocnemius medialis.LT-light touch, PP -pin prick
